# Evaluation of recombinant granule antigens GRA1 and GRA7 for serodiagnosis of *Toxoplasma gondii* infection in dogs

**DOI:** 10.1186/1746-6148-10-158

**Published:** 2014-07-12

**Authors:** Zedong Wang, Wei Ge, Si-Yang Huang, Jiping Li, Xing-Quan Zhu, Quan Liu

**Affiliations:** 1 State Key Laboratory of Veterinary Etiological Biology, Key Laboratory of Veterinary Parasitology of Gansu Province, Lanzhou Veterinary Research Institute, Chinese Academy of Agricultural Sciences, Lanzhou, Gansu Province 730046, PR China; 2 Military Veterinary Institute, Academy of Military Medical Sciences, Key Laboratory of Jilin Province for Zoonosis Prevention and Control, Changchun, Jilin Province 130122, PR China

**Keywords:** *Toxoplasma gondii*, GRA1, GRA7, Serodiagnostic marker, ELISA, Dogs

## Abstract

**Background:**

Toxoplasmosis, caused by the obligate intracellular parasite *Toxoplasma gondii*, is an important zoonotic disease worldwide. The precise detection of *T. gondii* infection in dogs has important public health significance. In this study, recombinant granule antigen proteins GRA1 and GRA7 were evaluated as potential diagnostic markers for *T. gondii* infection in dogs by an indirect enzyme-linked immunosorbent assay (ELISA).

**Results:**

GRA1 and GRA7 were cloned and expressed in *Escherichia coli*, and the recombinant GRA1, GRA7- and *Toxoplasma* lysate antigen (TLA)-based ELISAs were developed and evaluated using the canine positive and negative serum samples for anti-*T. gondii* antibodies determined by modified agglutination test (MAT) and indirect fluorescent antibody test (IFAT), showing a seroprevalence of 15.1% by TLA- and GRA1-ELISA, and 15.8% by GRA7-ELISA, and no significant difference was observed (*P >* 0.05). When compared with the two reference assays, MAT and IFAT, the GRA7-ELISA showed the highest co-positivity and co-negativity rates. Receiver operating characteristic (ROC) analysis revealed a largest area under curve (AUC) of 0.973 (95% CI, 0.955 to 0.991), and a highest relative sensitivity (93.2%) and specificity (94.0%) for a cut-off value of 0.809 in GRA7-ELISA.

**Conclusions:**

The results of the present study showed that GRA7-ELISA is highly sensitive and specific, and GRA7 is a potential serodiagnostic marker for the detection of *T. gondii* infection in dogs.

## Background

*Toxoplasma gondii*, an obligate intracellular parasite, can infect virtually all warm-blooded animals, including humans. Humans and animals become infected by eating undercooked or raw meat containing cysts, or by consuming food contaminated with sporulated oocysts [1]. Understanding the prevalence of *T. gondii* infection in dogs is of economic and public health importance. Firstly, the infection is serious in young dogs, especially in those co-infected with canine distemper virus [2]. Secondly, dogs are potentially involved in the mechanical transmission of *T. gondii* oocysts to humans, and shed parasite in their saliva [3-5]. Thirdly, in regions where dogs are used as food animals, *T. gondii* can be transmitted to humans by consumption of undercooked meat from infected dogs [6]. Lastly, dogs are the most common pets in the world, and also reflect the extent of *T. gondii* infection in the domestic environment [7].

Definitive diagnosis of *T. gondii* infection by mouse inoculation, or immunohistochemical analyses is optimal. However, these tests are time-consuming, involved in experimental animals, and may have a low sensitivity [8]. Multiple tests, such as indirect haemagglutination (IHA), modified agglutination test (MAT), latex agglutination test (LAT), indirect fluorescent antibody test (IFAT), and enzyme-linked immunosorbent assay (ELISA), are useful to demonstrate *T. gondii* infection in humans and animals. Despite the satisfactory results of serological tests, development of reliable and standard reagents remains a major constraint in serodiagnosis of *T. gondii* infection. Most conventional tests using tachyzoites grown in mice or in tissue culture are usually difficult to standardize, making the test results difficult to evaluate [9].

*T. gondii* dense granule antigen proteins (GRAs) are secretory proteins expressed by both tachyzoite and bradyzoite [10]. GRA1 is secreted into the parasitophorous vacuole (PV), which has Ca^2+^ binding domain, becoming a physiological important factor to invade in the host cells [11], and GR7 is secreted into the cytoplasm of bradyzoite-infected cells and within the PV and the PV membrane in tachyzoite-infected cells [12]. GRA1-based ELISA shows a sensitivity of about 60%, but the specificity can reach 98% in humans [13,14]. GRA7 based-ELISA has presented overall specificity of 98 to 100% and sensitivity of 81 to 88% in humans and goats [15,16]. Moreover, GRA7-ELISA has the highest positive rate in pregnant women, compared with the rhoptry (ROP1), matrix antigens (MAG1), the major surface antigen (SAG1), and GRA8 [17]. However, there are few reports on evaluation of GRAs as potential diagnostic markers for *T. gondii* infection in dogs. In the present study, recombinant proteins GRA1 and GRA7 were expressed and evaluated for serodiagnosis of *T. gondii* infection in dogs by indirect ELISA.

## Methods

### Ethics statement

The pet dogs from which blood were collected, were handled in accordance with good animal practices required by the Animal Ethics Procedures and Guidelines of the People’s Republic of China. The client owned pet dogs were admitted into pet hospitals in Lanzhou City, Gansu province, Northwest China. Consent was obtained from the owners of the pet dogs. The present study was approved by the Animal Ethics Committee of Lanzhou Veterinary Research Institute, Chinese Academy of Agricultural Sciences (Approval No. LVRIAEC2010-005).

### Preparation of *Toxoplasma* lysate antigen (TLA)

*T. gondii* soluble antigens (TLA) was prepared by sonicating the purified *T. gondii* tachyzoites of GT1 strain, and diluted to a final concentration of 1 mg/ml in PBS as described elsewhere [18].

### Expression and purification of recombinant GRA1 and GRA7

Based on the nucleotide sequence of GRA1 (HM067753) and GRA7 (JX045574), the PCR primers for amplification of the gene products of 570-bp GRA1 (forward: 5′-AA*CCATGG*TGCGTGTGAGCGCTATTG-3′; reverse: 5′-GC*GAATTCC*CTCTCTCTCTCCTGTTAGGAAC-3′) and 675-bp GRA7 (forward: 5′-ATT*CCATGG*CGGCCACCGCGTCAGAT-3′; reverse: 5′-GC*GAATTCC*TCTTCTGTGTCTGTCTGCCTCTC-3′) were designed. The italics in the primer sequences represented the *Eco*RI and *Nco*I linker sites in the expression vector pET-28a (Novagen). The PCR was performed at an annealing temperature of 58˚C for 45 s. The resulting gene products were cloned into the *Eco*R1/*Nco*I site of pET-28a to generate a recombinant plasmids pET28-GRA1 and pET28-GRA7, which were confirmed by restriction enzymes and sequencing, and further processed for the expression of recombinant products in *Escherichia coli* BL21 (DE3) according to the standard techniques.

The recombinant proteins were analyzed by sodium dodecyl sulfate-polyacrylamide gel electrophoresis (SDS-PAGE) using a 12% polyacrylamide gel. The reactivity with *T. gondii* positive sera was tested by immunoblot. Blots of recombinant GRA1 and GRA7 were incubated with *T. gondii*-positive mice sera, followed by alkaline phosphatase-conjugated goat anti-mouse antibodies. The recombinant GRA1 and GRA7 were purified using a Ni-NTA purification system (Qiagen, Germany) according to the manufacturer’s protocol.

### Serum samples

A total of 259 blood samples were collected from dogs between November and December 2010 in Lanzhou, China. Sera were separated by centrifugation at 1,500 × g for 10 min and stored at -20°C until use. The positive and negative samples for *T. gondii* infection were determined by MAT and IFAT [19-21].

### ELISA

Indirect ELISA was performed to test *T. gondii* infection in dogs as described by elsewhere [22]. Briefly, microplates were coated with TLA, GRA1, or GRA7, respectively. After washing, 100 μl of canine serum diluted 1:50 was added to each well, and incubated for 3 h at 37°C, then 100 μl of horseradish peroxidase-conjugated sheep anti-dog IgG antibodies (Abcam, USA) diluted 1:20,000 was added. After incubation for 1 h at room temperature and washing, color was developed by the addition of a substrate solution containing tetramethylbenzidine chromogenic substrate TMB (Thermo Fisher Scientific, USA), and stopped by 2 M H_2_SO_4_. The optical densities (ODs) were measured at 450 nm in a microplate reader (BioTek, USA). ELISA results were determined for each serum in duplicate. The cut-off point of OD values of a positive samples was set to be at least two times higher than that of the negative samples at any dilution point.

### Data analysis

The significance of association between the results of ELISAs was analyzed using the McNemar chi-square test. The degree of agreement between the results from the 2 tests was quantified using Kappa statistics. Sensitivity and specificity of ELISA was determined as described by elsewhere [23]. The expected performance of the ELISAs at different cut-off points was examined using the receiver operating characteristic (ROC) curves [24].

## Results

### Expression and purification of recombinant *T. gondii* GRA1 and GRA7

**Figure 1:**
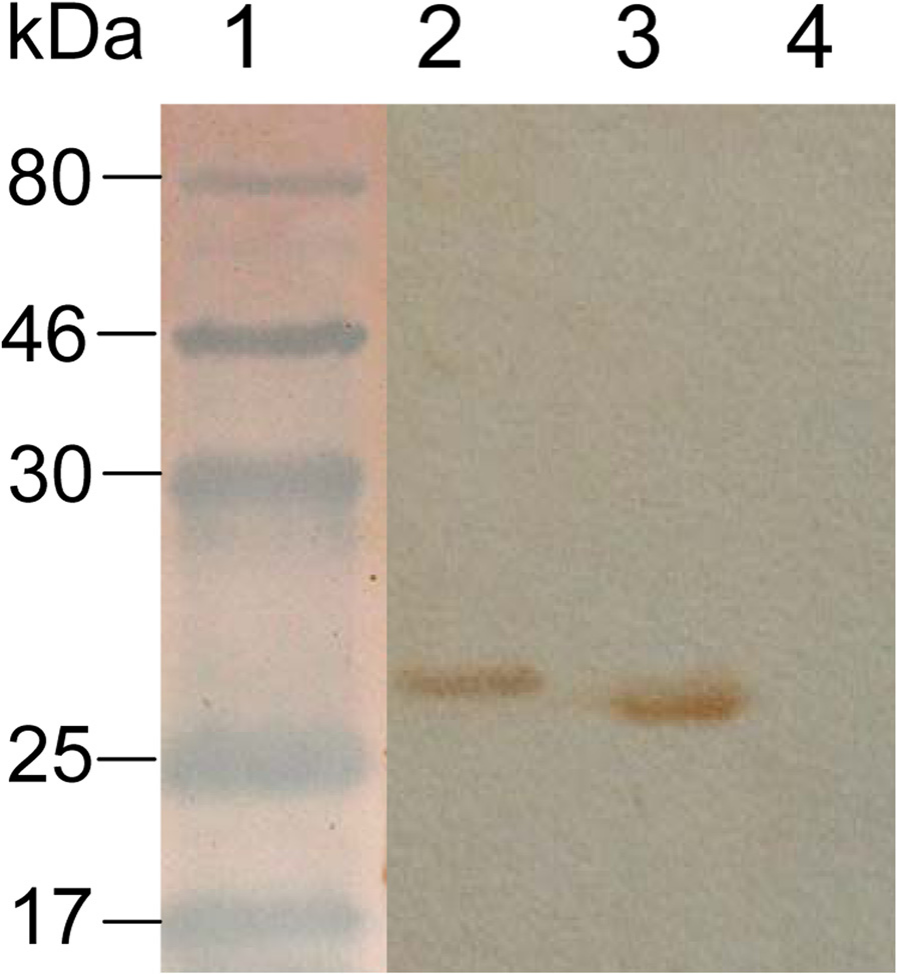
**Western blotting analysis of the recombinant GRA1 and GRA7 expressed in *E. coli*.** Lane 1: molecular protein marker; Line 2: the expressed GRA7; Line 3: the expressed GRA1; Line 4: control culture of cells lacking the GRA1 or GRA7 insert.

The GRA1 and GRA7 encoding genes were amplified, and cloned into expression vector pET-28a to construct recombinant plasmids pET28-GRA1 and pET28-GRA7, which were transformed into *Escherichia coli* BL21 (DE3), and induced by IPTG. The immunoreactivity of the expressed proteins was confirmed by Western blot analysis using the mice sera positive for anti-*T. gondii* IgG antibodies (Figure 1). The recombinant proteins GRA1 and GRA7 were purified using a Ni-NTA purification system, showing the purity of more than 95%.

### Evaluation of the diagnostic performance of TLA, GRA1 and GRA7

To evaluate the potential of recombinant proteins for serodiagnosis of *T. gondii* infection in dogs, three separate ELISAs were developed using TLA, GRA1 and GRA7 as coating antigens. The optimal working dilution, determined by checkerboard assays using serial dilutions of antigens and sera, was shown 10 μg/ml for TLA, and 5 μg/ml for both GRA1 and GRA7.

The canine serum samples were detected for anti-*T. gondii* antibodies by MAT and IFAT, showing that there were 44 positive samples and 215 negative samples. These samples were tested by TLA-, GRA1-, and GRA7-ELISA, respectively. As shown in Table 1, there were 42 positive and 217 negative samples by TLA- and GRA1-ELISA, while there were 44 positive and 215 negative samples tested by GRA7-ELISA (Table 1), demonstrating that the seroprevalence was 16.2% by TLA- and GRA1-ELISA, and 17.0% by GRA7-ELISA (Table 2). There was no significant difference between positive and negative results when comparing TLA-ELISA with GRA1-, and GRA7-ELISA results using McNemar chi-square (*P >* 0.05). A substantial agreement (90.7%) was found between TLA- and GRA1-ELISA (κappa = 0.66; 95% confidence interval [CI], 0.53 to 0.79), and a perfect agreement (94.6%) was observed between TLA- and GRA7-ELISA (κappa = 0.80; 95% confidence interval [CI], 0.71 to 0.90).

**Table 1:** **Detection results of *Toxoplamsa gondii *** **antibodies in dogs by ELISA based on TLA, GRA1 and GRA7**

	**MAT and IFAT**		
	**No. of positive**	**No. of negative**	**Total**
TLA-ELISA			
No. of positive	37	5	42
No. of negative	7	210	217
Total	44	215	259
GRA1-ELISA			
No. of positive	34	8	42
No. of negative	10	207	217
Total	44	215	259
GRA7-ELISA			
No. of positive	39	5	44
No. of negative	5	210	215
Total	44	215	259

**Table 2:** **Prevalence of *Toxoplasma gondii *** **antibody and the titers in dogs by ELISA based on TLA, GRA1 and GRA7**

**Detection methods**	**No. examined**	**No. (%) of positive**	**No. (%) of samples showing the antibody titers at**		
			**1:50**	**1:100**	**≥1:200**
TLA-ELISA	259	42 (16.2)	25 (9.7)	8 (3.1)	9 (3.5)
GRA1-ELISA	259	42 (16.2)	25 (9.7)	7 (2.7)	10 (3.9)
GRA7-ELISA	259	44 (17.0)	26 (10.0)	9 (3.5)	9 (3.5)

When compared with the two reference assays, MAT and IFAT, 5 false positive and 7 false negative samples were found in TLA-ELISA, 8 false positive and 10 false negative samples were found in GRA1-ELISA, and 5 false positive and 5 false negative samples were found in GRA7-ELISA (Table 1), demonstrating that the GRA7-ELISA showed the highest co-positivity and co-negativity rates (Table 3).

**Table 3:** Comparison of diagnostic performance of ELSIA based on TLA, GRA1 and GRA7

**Detection method**	**Sensitivity (%)**	**Specificity (%)**	**False positive rate (%)**	**False negative rate (%)**
TLA-ELISA	88.1	96.8	11.9	3.2
GRA1-ELISA	81.0	95.4	19.0	4.6
GRA7-ELISA	91.0	97.7	9.0	2.3

### ROC analysis

**Figure 2:**
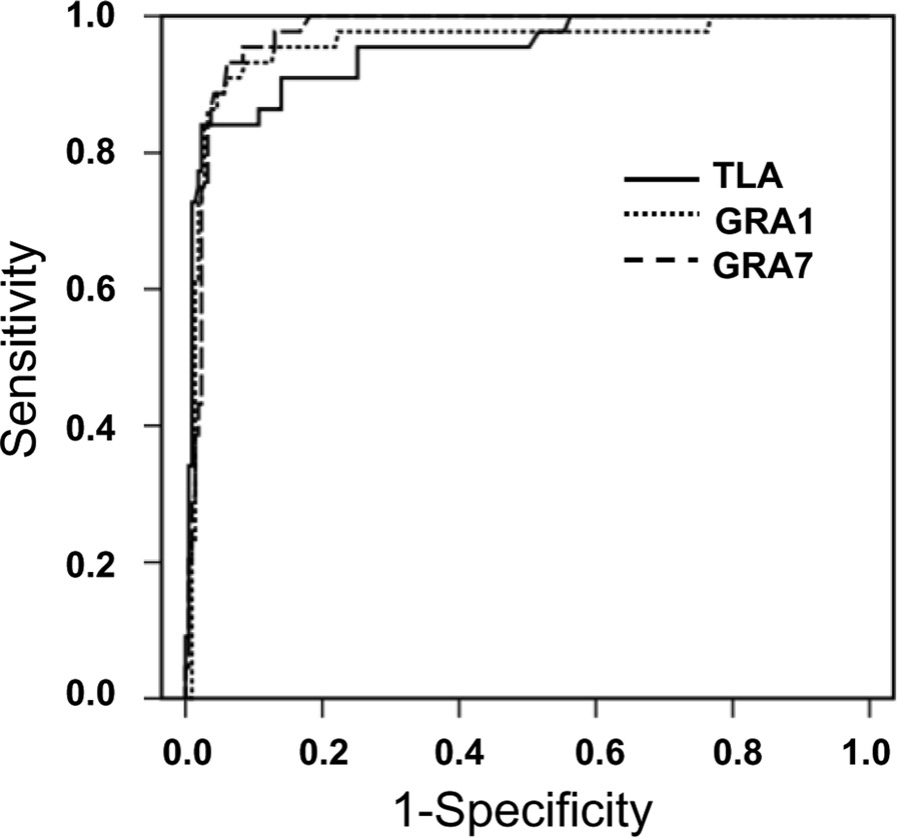
**Receiver operating characteristics (ROC) analysis of the ELISAs.** ROC analysis showed an area under the curve (AUC) of 0.948 (95% CI, 0.911 to 0.986) for TLA-ELISA, 0.957 (95% CI, 0.919 to 0.995) for GRA1-ELISA, and 0.973 (95% CI, 0.955 to 0.991) for GRA7-ELISA, respectively.

**Figure 3:**
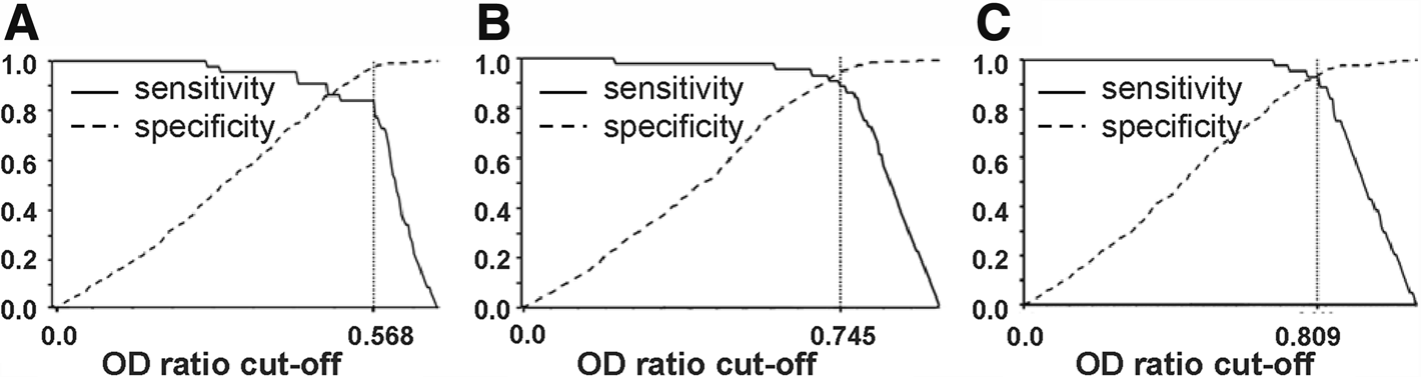
**Relative sensitivity and specificity of TLA- GRA1 and GRA7-ELISA at different cut-offs. (A)** Vertical line shows the selected cut-offs of 0.568 for TLA-ELISA. **(B)** Vertical line shows the selected cut-offs of 0.745 for GRA1-ELISA. **(C)** Vertical line shows the selected cut-offs of 0.809 for GRA7-ELISA.

ROC analysis revealed an area under curve (AUC) of 0.948 (95% CI, 0.911 to 0.986) for TLA-ELISA, 0.957 (95% CI, 0.919 to 0.995) for GRA1-ELISA, and 0.973 (95% CI, 0.955 to 0.991) for GRA7-ELISA, respectively (Figure 2). The estimated sensitivities and specificities for different OD ratio cut-off values were obtained from ROC analysis (Figure 3), indicating that the cut-off value at 0.568 for TLA-ELISA shows a sensitivity of 84.1% and a specificity of 97.7%, and 0.745 for GRA1-ELISA shows a sensitivity of 91.0% and a specificity of 94.0%, and 0.809 for GRA7-ELISA shows a sensitivity of 93.2% and a specificity of 94.0%, which were considered as the most appropriate cut-off for the 3 tests.

## Discussion

Diagnosis of toxoplasmosis by demonstration of *T. gondii* is usually difficult. Bioassays using mice or cats are not suitable for rapid detection or field application, due to their price, sensitivity and the length of time to reach the results. Serological tests are still considered useful for evaluation of the infection in humans and animals. IHA, MAT, LAT, IFAT and ELISA have been used to detect *T. gondii* infection in dogs [4,25-28]. However, the sensitivity and specificity of these tests may vary between sampling methods, or reference populations, even when the same tests are used in different species. IHA has poor sensitivity and concordance when compared with ELISA, precluding their use for detection of *T. gondii* infection in dogs [29]. MAT is the most widely used methods for detection of *T. gondii* infection worldwide, as it is not species specific. However, MAT shows high false negative rate, and low agreement in dogs compared with IFAT and ELISA [20,30]. IFAT is one of the most widely used methods for detecting anti-*T. gondii* antibodies, but it is conduct only in reference laboratories, or by laboratory technicians that have enough experience for this test [31]. Thus, ELISA is recommended to detect *T. gondii* infection in dogs [30].

Most commercial ELISAs using native tachyzoite antigens may vary significantly between laboratories, or between batches. An alterative approach is to develop serological tests using recombinant proteins, with an advantage of the precise antigen composition, and easy standardization [32]. Many recombinant antigens have been produced and evaluated for serodiagnosis of *T. gondii* infection in humans and animals, including surface antigens (SAG1, and SAG2), rhoptry proteins (ROP2, and ROP4), microneme proteins (MIC1, and MIC3), and dense granule antigens (GRA1, GRA2, GRA5, GRA6, GRA7, and GRA8) [32,33]. Among these antigens, GRA1 is a major secretory antigen in chronic infection, and GRA7 induces a very strong antibody response in the acute infection [33]. In the present study, recombinant GRA1 and GRA7 were evaluated for serodiagnosis of *T. gondii* infection in dogs by indirect ELISA, whose results were compared with TLA-ELISA. A substantial agreement (>90.0%) was observed among the 3 tests. If the detection results of MAT and IFAT were used as references, GRA7 showed high sensitivity and specificity as well as low FPR and FNR in comparison to GRA1 and TLA, suggesting that GRA7 is a good serological marker for the detection of *T. gondii* infection in dogs.

GRA7 is expressed by all developmental stages, including tachyzoites and bradyzoites, and is abundant on the surface and cytoplasmic matrix of host cells, the PVM, and within the PV lumen [17]. GRA7 can be used to detect anti-*T. gondii* antibodies in chronic and acute infection, but it is more associated with acute infection [34,35]. In humans infected with *T. gondii*, GRA7 appears significantly earlier than other antigens, such SAG1 and MAG1 [36,37]. When GRA7 is released from tachyzoites and bradyzoites, it has direct contact with the host immune system, and induces strong antibody responses in both early and late stages of infection [12,15]. The antigenic properties of GRA7 make it a good serological maker for the detection of anti-*T. gondii* antibodies in chronic and acute infection.

ROC analysis enabled us to compare the relative sensitivity and specificity of the developed ELSIAs at various cut-offs, in which the AUC represents a statistical summary of the overall diagnostic performance of the test [38,39]. In this study, the AUC of 0.973 for GRA7-ELISA suggests that it represents a highly accurate test with good discrimination of positive from negative samples, and the cut-off at 0.809 showed the most appropriate sensitivity of 93.2% and specificity of 94.0%, respectively.

## Conclusion

The present study, for the first time, evaluated the dense granule antigens GRA1 and GRA7 as potential diagnostic markers for *T. gondii* infection in dogs by ELISA, showing that the GRA7-ELISA is highly sensitive and specific, and that GRA7 is a potential serodiagnostic marker for the detection of *T. gondii* infection in dogs.

## Competing interests

The authors declare that they have no competing interests.

## Authors’ contributions

QL and XQZ conceived and designed the study. ZW, WG and SYH performed the experiments, analyzed the data. JL helped in the study design and manuscript revision. QL and XQZ wrote the manuscript. All authors read and approved the final manuscript.
